# Zinc Transporters and Zinc Signaling: New Insights into Their Role in Type 2 Diabetes

**DOI:** 10.1155/2015/167503

**Published:** 2015-04-23

**Authors:** Stephen A. Myers

**Affiliations:** University of Tasmania (UTAS), School of Health Sciences, Newnham Campus, Launceston, TAS 7250, Australia

## Abstract

Zinc is an essential trace element that plays a vital role in many biological processes including growth and development, immunity, and metabolism. Recent studies have highlighted zinc's dynamic role as a “cellular second messenger” in the control of insulin signaling and glucose homeostasis. Accordingly, mechanisms that contribute to dysfunctional zinc signaling are suggested to be associated with metabolic disease states including cancer, cardiovascular disease, Alzheimer's disease, and diabetes. The actions of the proteins that control the uptake, storage, and distribution of zinc, the zinc transporters, are under intense investigation due to their emerging role in type 2 diabetes. The synthesis, secretion, and action of insulin are dependent on zinc and the transporters that make this ion available to cellular processes. This suggests that zinc plays a previously unidentified role where changes in zinc status over time may affect insulin activity. This previously unexplored concept would raise a whole new area of research into the pathophysiology of insulin resistance and introduce a new class of drug target with utility for diabetes pharmacotherapy.

## 1. Introduction

Type 2 diabetes (T2D) is a disease that is largely associated with increased rates of obesity and reduced physical activity [1]. It is a common metabolic disorder that is characterized by chronic hyperglycemia coupled with reduced life expectancy resulting from debilitating disease states that include heart disease, stroke, peripheral neuropathy, and renal disease [2]. The etiology of TD2 reflects its multifaceted and complex interactions with the environment, genetics, and lifestyle resulting in a necessitated multipronged approach towards better management and treatment options. Moreover, habitual changes in lifestyle and nutrition to better manage the symptoms associated with T2D have proven somewhat successful in the short term [3], but most patients usually find it difficult to maintain these strategies in the long term due to differing experiences or perceptions [4]. Accordingly, opportunities to develop better long-term therapies are greatly needed to ameliorate the symptoms and clinical features of this disease.

In this context, research underpinning the mechanisms of T2D has revealed a novel role for zinc in insulin signaling. Zinc has “mimetic” activity where it is involved in a range of functions including insulin receptor signal transduction, insulin storage, secretion and tissues/organelle distribution, and inhibition of protein tyrosine phosphatases [5–8]. In insulin-dependent peripheral tissues such as skeletal muscle, adipose, and liver, zinc ions play a role in insulin-induced glucose transport and glycemic control [9–16]. Thus, the proteins that transport zinc likely facilitate cell signaling processes that contribute to glycemic control in peripheral tissues by modulating cytosolic zinc concentrations. For example, aberrant subcellular signaling of zinc concentrations in the cytosol and organelles may contribute to insulin responsiveness [17] and thus promote insulin resistance. However, questions on how zinc transporters are regulated and effectively facilitate zinc flux contributing to cell signaling are largely unknown.

## 2. Zinc

Zinc is a trace element that is critically important for the growth and development of microorganisms, plants, and animals [18]. In humans, zinc is found in all body tissues and secretions contributing to approximately 2–4 g of total zinc in the adult body [18, 19] and is therefore the most abundant trace metal in tissue next to iron of which there is approximately 4 g localized mostly in blood [15]. In tissues, zinc concentration is highest in the prostate (approximately 200 *μ*g/g), then pancreas (approximately 140 *μ*g/g), and muscle (approximately 50 *μ*g/g), while in plasma there is approximately 14–16 *μ*M of total zinc that is distributed to cells [19]. At the cellular level, total zinc concentrations of human cells are 200–300 *μ*M [20]. Given the low plasma concentration of zinc and its importance in cellular signaling, it is essential that the availability and distribution of “free” zinc (free zinc is used to differentiate zinc involved in cell signaling from zinc that tightly bound to protein and therefore thermodynamically unavailable) are tightly controlled [6].

### 2.1. Identification of Free Zinc

Early efforts to identify the concentration of “free” zinc in cells and organelles were hindered due to the lack of specific imaging technologies and detection reagents [21]. However, recently efforts to describe “free” zinc in cells and subcellular organelles have relied on synthetic and biochemical approaches that provide (1) specific binding of zinc over other competing ions such as Ca^2+^, H^+^, or Fe^2+^; (2) a rapid and reversible response over a wind range of zinc concentrations; and (3) stability, solubility, and reduced toxicity [22]. Although there are several novel technologies for measuring zinc concentrations in various cells and cellular organelles, there are inconsistencies depending on the type of biological samples and detection methods used. For example, zinc concentrations* in vitro* are significantly different from those* in vivo*. This is due mostly to the nonphysiological metal-buffering capacity in media such as that found in fetal bovine serum and serum-free media versus the physiological buffering capacity of whole blood [20].

Another factor involved in the accuracy of measuring “free” zinc in cells depends on the type of analytic sensor/protein probe used. There are mainly two types of technologies used for the detection of “free” zinc in cells: low molecular weight (LMW) fluorescent/fluorogenic chelating agents (probes) and genetically encoded fluorescent proteins (sensors) [20]. The LMW fluorescent/fluorogenic probes differ in their selectivity and specificity. For example, studies using Zinpyr-1, FluoZin-3 AM, Newport Green DCF, and Zinquin ethyl ester probing for “free” zinc in bone marrow macrophage cell lines found that Zinpyr-1 fluoresced effectively in response to “free” zinc, but the other probes weakly or negligibly responded [23].

The analytical measurement of “free” zinc in the cytosol has been attempted using both LMW probes and protein sensors. Both have resulted in large differences in the estimated concentration of “free” zinc ranging from *μ*M [24] to 100–300 pM [25] using LMW probes and from 5–10 pM [26] to 180 nM using protein sensors [27]. Although it has been observed that there are many inherent issues associated with measuring “free” zinc in cells, the technology has given much needed temporal and spatial resolution of cellular zinc localization, compartmentalization, and distribution [20]. In this context, the compartmentalization and distribution of zinc are tightly controlled so that cellular zinc homeostasis is maintained within an appropriate physiological range. This is achieved by a class of proteins called the metallothioneins [28] and a family of zinc transporter proteins [15, 17, 29–35].

## 3. Zinc Transporters

**Figure 1:**
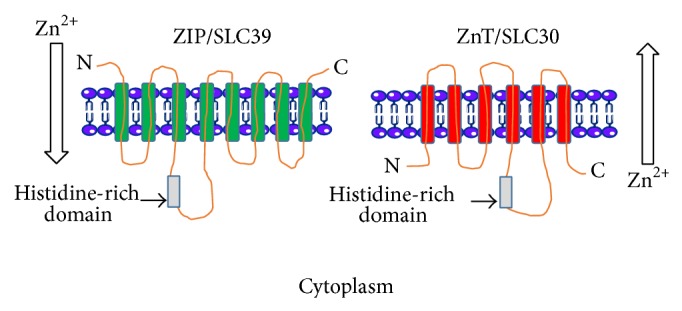
The predicted membrane topologies of the zinc transporters ZIP/SLC39 and ZnT/SLC30. ZIP/SLC39 has eight predicted transmembrane domains (TMDs) and a large histidine-rich domain between TMD3 and TMD4. The predicted number of TMDs for ZnT/SLC30 is six, with a large histidine-rich region between TMD4 and TMD5. The direction of zinc flux in relation to the cytoplasm is shown. Figure was adapted from Eide (2006) [30] and the image was produced using Servier Medical Art: http://www.servier.com/.

Zinc transporters belong to a family of transmembrane proteins that control the flux of zinc across cellular membranes and therefore contribute to the distribution, storage, and compartmentalization of zinc [15]. Zinc transporters belong to two major gene families: the ZnT proteins (solute-linked carrier 30, SLC30) and the ZIP (Zrt/Irt-like, solute-linked carrier, 39, SLC39) [31–33]. In mammals, there are ten members of the zinc efflux transporters (ZnT1–10) and fourteen members of the zinc influx transporters (ZIP1–14). The ZIP proteins transport zinc into the cell via the plasma membrane or out of subcellular organelles when cytosolic zinc is low or depleted, while the ZnT proteins transport zinc out of cells via the plasma membrane or into subcellular organelles when zinc concentrations are high [17, 29–34, 36] (Figure 1).

### 3.1. ZIP/SLC39 and ZnT/SLC30 Family

The ZIP (for consistency, ZIP and ZnT will be used for the zinc transporters SLC39 and SLC30, resp.) family of zinc transporters have eight predicted transmembrane domains (TMDs) with an extracytosolic N- and C-terminal (Figure 1). A long histidine-rich loop region is located between TMD3 and TMD4 [37] and is thought to bind metal ions [38]. The ZIP family is expressed in a wide range of tissues and cells and their proteins are located to distinct cellular compartments. For a comprehensive review, see Jeong and Eide [37] and references therein. Similar to the ZIP transporters, members of the ZnT family have a topology of six predicted transmembrane domains and a histidine-rich loop region between TMD4 and TMD5 and the N- and C-termini are located on the cytosolic side of the membrane [39, 40] (Figure 1). The ZnT family is also expressed in a wide range of tissues and cells and has distinct subcellular locations. For a comprehensive review, see Huang and Tepaamorndech [41] and references therein.

## 4. Zinc, Zinc Transporters, and Cell Signaling

### 4.1. Zinc and Cell Signaling

The critical importance of maintaining zinc concentrations and cellular homeostasis is highlighted by the diverse number of zinc transporters controlling zinc flux. The processes of cellular signaling are complex, and although there are abundant examples in the literature of proteins involved in signal transduction, the role of zinc and zinc transporters in cellular signaling is less defined.

Zinc mimics the action of hormones, growth factors, and cytokines and given the large number of zinc transporters dedicated to controlling zinc homeostasis it is not surprising that this ion is taking precedence as a leading cell signaling molecule analogous to calcium. The mechanisms of zinc's insulin-mimetic activity have been clearly shown in studies on glucose metabolism [13, 16, 42–44] and lipid metabolism [11, 45]. For example, zinc inhibits protein tyrosine phosphatases (PTPs), an important class of enzymes implicated in dephosphorylation [7, 8, 12, 46, 47]. Thus, in the context of insulin signaling, the inhibition of PTPs by zinc facilities net phosphorylation of the insulin receptor, thereby promoting the activation of its signaling cascade [8, 47]. In fact, the zinc-mimetic effect on cellular homeostasis is numerous and includes the stimulation of glucose uptake and lipogenesis in adipocytes [13, 48], tyrosine phosphorylation of the insulin/IGF-1 receptor and insulin receptor substrate-1 [7, 47], activation of the epidermal growth factor receptor [49, 50], inhibition of PTPs [7, 8, 47], activation of the transcription factor FOXO1a and the key gluconeogenic regulatory enzymes phosphoenol pyruvate carboxykinase (PEPCK) and glucose 6-phosphatase (G6Pase) [51], and the activation of mitogen-activated protein kinases (MAPKs): extracellular signal regulated kinases 1 and 2 (ERK1/2), c-Jun N-terminal kinase (JNK), and p38 [52].

### 4.2. Zinc Transporters and Cell Signaling

Given the known role of zinc transporters in mediating zinc flux into and out of cells and subcellular organelles, we can begin to gain some understanding of their importance in metabolic processes from studies that have addressed their function in cell and animal models. Studies addressing the role of overexpressed ZnT7 in pancreatic insulinoma RIN5mf cells identified that glucose stimulation of these cells led to a significant increase in insulin secretion and subsequent insulin biosynthesis [53]. Similarly, ZnT7 null mice were more susceptible to diet-induced glucose intolerance and insulin resistance [54].

Studies by Smidt et al. (2009) [55] found that other ZnT family members, ZnT3, ZnT5, and ZnT8, were differentially regulated by glucose (range of glucose concentrations: 2 mM, 5 mM, and 16 mM over 24-hour stimulation) in INS-1E cells [55]. Similarly, while Bellomo et al. (2011) showed a significant increase in the zinc transporters ZIP6–8 on the addition of 16.7 mM glucose over 24 hours in mouse pancreatic islets, there was no increase in ZnT3, ZnT5, or ZnT8 [56] and this disagrees with the studies done by Smidt et al. [55]. This may reflect the use of CD1, 12-week-old female mice used by Bellomo et al. [56] versus 4-week-old male BALB/CA mice used by Smidt et al. [55]. Evidently, further studies in a range of tissue and cell types are required to determine the effect (if any) of glucose on the expression of the zinc transporters.

Studies on streptozotocin-treated mice having ZnT3 knock-out observed that these mice displayed decreased insulin secretion and higher glucose levels and had clinical hyperglycemia [55]. However, this study should be treated with caution due to the fact that these observations were only apparent after the streptozotocin treatment and therefore possible differences in streptozotocin effectiveness to destroy the beta cells should be taken into account.

Fasting gluconeogenesis is impaired in the livers of ZIP14 null mice which are attributable to G-protein coupled receptor impairment [57]. Moreover, ZIP14 null mice have greater body fat, are hypoglycemic, and have increased levels of insulin.

Recently we have identified a role for ZIP7 in glycemic control in skeletal muscle. We have shown that the reduction of ZIP7 by siRNA in C2C12 mouse skeletal muscle cells is associated with a reduction of several genes implicated in glycogen metabolism including the insulin receptor (IR), IR-substrate 1 and 2, Glut4, and glycogen branching enzyme (Gbe) [58]. This was concomitant with reduced Glut4 protein, reduced glycogen synthesis, and reduced phosphorylation of AKT and suggests that ZIP7 controls glycogen synthesis in these cells via the phosphorylation of AKT and Glut4 mobilization. Supporting evidence for these studies comes from recent studies in TamR MCF-7 breast cancer cells and lymphocyte cells [59, 60]. In TamR MCF-7 cells it was found that ZIP7 was phosphorylated by casein kinase 2 and this was associated with the “gated” release of zinc from intracellular stores leading to the activation of tyrosine kinases and the phosphorylation of AKT and extracellular signaling kinases 1 and 2 [59]. In ZIP9 deficient DT40 B lymphocyte cells it was observed that AKT and ERK phosphorylation was reduced concomitant with an increase in PTP activity [60]. Furthermore, overexpression of ZIP9 in the DT40 deficient cells restored AKT and ERK phosphorylation and decreased PTPase activity in response to zinc treatment [60].

## 5. Zinc Transporters and T2D

T2D is a highly complex disease characterized by insulin resistance and impaired glucose homeostasis. Recently, emerging evidence has suggested a role for zinc and zinc transport proteins in facilitating glycemic control and glucose homeostasis [58]. Given the increase in the global incidence of T2D [61], there is considerable interest in understanding the molecular mechanisms of zinc transport and zinc action on cellular pathways associated with glucose metabolism. In the context of dietary zinc supplementation to ameliorate diabetes complications in both animal models and humans, there have been many contradictory results on zinc's efficacy [19, 62–69]. While these studies on zinc supplementation as an adjunct therapy to treat diabetes are ongoing, the transporters implicated in controlling zinc flux in cells and their role in diabetes are limited.

### 5.1. ZnT8 and Diabetes in Humans

One of the most highly explored zinc transporters in diabetes is ZnT8. This transporter is almost exclusively expressed in the beta cells of the pancreas where it plays a critical role in the transport of zinc into insulin secretory vesicles and is fundamental for the synthesis, storage, and action of insulin [29–35, 70–75]. It has been shown that the overexpression of ZnT8 in INS-1 cells induces glucose-stimulated insulin secretion [76]. In contrast, reduced expression of this ZnT8 in INS-1 cells resulted in reduced insulin concentrations and reduced insulin secretion in response to a hyperglycemic stimulus [77]. Similarly, it has been shown that mice with a beta cell pancreatic-specific ZnT8 knockout have glucose intolerance [78], while global ZnT8 null mice have abnormalities in diet-induced glucose tolerance and insulin secretion [74, 79] and have exacerbating diet-induced obesity resulting in insulin resistance [74, 80].

ZnT8 is suggested to be associated with the risk of T2D in humans due to a nonsynonymous SNP (rs13266634 C>T) which exchanges arginine (R) with tryptophan (W) at amino acid position 325 in European and Chinese patients [81–84]. The SNP disrupts a protein kinase A and protein kinase C recognition motif that compromises ZnT8 functionality [85] in transporting zinc ions into the secretory granule where insulin is matured and stored in its hexameric form bound to zinc ions [74]. ZnT8 is also suggested to be an autoantigen due to studies that identified autoantibodies to this transporter in 60–80% of new onset cases of 223 type 1 diabetes patients [86]. However, recently a study by Flannick et al. (2014) sequenced or genotyped approximately 150,000 individuals across five ethnicities and identified 12 rare, predicted ZnT8 protein truncation variants that conferred a 65% reduced risk of T2D [87]. Moreover, in nondiabetic Icelandic carriers that had a frameshift variant (p.Lys34SerfsX50), it was demonstrated that these individuals had reduced glucose levels [87]. Of the 12 variants, the most common two identified (p.Arg138X and p.Lys34SerfsX50) encoded unstable ZnT8 proteins and were associated with T2D protection [87]. Accordingly, these authors suggest that inhibition of ZnT8 might prove to be a useful therapy for the treatment of T2D.

### 5.2. Other Zinc Transporters and T2D in Humans

Because ZnT8 has had such a significant presence in diabetes research, other transporters have not had the same focused attention until only recently. For example, Foster et al. (2014) [88] investigated the effect of zinc supplementation and flaxseed oil on the gene expression of zinc transporters, metallothioneins (MTs), and markers of glycemic control in peripheral blood mononucleocytes (PBMCs) of 48 postmenopausal women with T2D [88]. Over 12 weeks of experimentation there were no significant effects of zinc or flaxseed oil supplementation on glycemic markers, MTs (MT-1A and MT-2A), or zinc transporters ZnT1, ZnT5–ZnT8, ZIP1, ZIP3, ZIP7, and ZIP10. Although the information from these studies highlights the expression of zinc transporters in PBMCs, the study should be analyzed with caution. For example, how does the expression of zinc transporters in PBMCs relate to glycemic control given that a number of the zinc transporters are tissue-specific? In this context, ZnT8 is almost exclusively expressed in the beta cells of the pancreas where it plays a critical role in transporting zinc from the cytoplasm into insulin secretory vesicles, an important step in insulin synthesis and secretion [75]. Similarly, Zip7 is mostly expressed in the Golgi apparatus and the endoplasmic reticulum and has been designated as the “gate-keeper” of zinc release into the cytosol where it is involved in cell signaling events [50, 59].

Apart from research in animal models and cell culture systems (see zinc, zinc transporters, and cell signaling) there is little information available on the role of other members of the zinc transporter family in human T2D.

## 6. Therapeutic Utility of Zinc and Zinc Transporters

The relevance of intracellular zinc signaling in animal and cell models has been recently highlighted. Although several studies have pointed to zinc as an adjunct therapy in the treatment of zinc-deficient T2D patients, the cellular mechanisms that lead to dysfunctional zinc signaling and/or partitioning, rather than zinc deficiency, will require the development of novel drugs that target zinc transporters for example. Moreover, there are several issues associated with zinc supplementation for T2D patients such as the undesirable effects of elevated HbA1c and high blood pressure [66]. Furthermore, while zinc has insulin mimetic effects, its clinical application for lowering and improving glycemic control in patients with T2D is complicated by the fact that zinc absorption rates are low and high doses and long-term zinc supplementation are required [66].

In this context, several zinc complexes have been developed to address these complexities (see [66], for a comprehensive review). The opportunities for translating zinc-complexes into clinically relevant applications will require intensive investigation into the properties of these zinc complexes, their absorption rates, tissue distribution, toxicity, insulin-mimetic activity, and efficacy in ameliorating blood glucose levels in human T2D.

Given our current understanding on the role of zinc as a critical modulator of cellular signaling and homeostasis and the fact that the storage, compartmentalization, and cellular distribution of zinc are tightly regulated by zinc transporters, it is reasonable that dysregulation of zinc flux will play an important role in disease processes. Moreover, the processes of zinc-related cellular dysfunction are mostly due to alterations in the proteins that control zinc flux and homeostasis rather than an issue of zinc deficiency or accumulation. Accordingly, drugs that target zinc transporters may provide utility to correct or improve dysfunctional zinc homeostasis and could therefore be more efficient than zinc supplementation. Although zinc is implicated in cellular signaling events, several questions remain to be resolved. For example, what extracellular stimulus regulates the expression of the zinc transporters? Are the zinc transporters differentially regulated in a tissue- or cell-specific manner? How does each specific zinc transporter regulate cellular signaling pathways? Moreover, genetic studies such as the targeted, tissue-specific disruption of these transporters in animal models will assist in determining the function of these proteins.

## 7. Conclusion

Zinc is an essential trace element that is implicated in numerous normal and pathophysiological cellular functions. The emerging role of zinc as an insulin mimetic and the ubiquitous nature of this ion in maintaining cellular function suggest that abnormal cellular partitioning and levels of zinc will have biological and clinical effects. Although our current understanding on the role of zinc transporters in T2D is limited, it is clear from studies on ZnT8 that this family of transporters has utility for the development of novel diabetic therapies. While ZnT8 plays a significant role in insulin biology and therefore represents an attractive target for diabetes therapy, the other members of the zinc transporter family in diabetes are less defined. However, we can speculate from the information presented in this review that the other transporters are involved in processes that facilitate insulin signaling and glycemic control and therefore could offer exciting new targets that are amendable to therapeutic intervention in the treatment of diseases associated with insulin resistance and T2D.
